# *Nostoc* sp. extract enhances rice lodging resistance by remodeling the gene expression network: mechanism and field application

**DOI:** 10.1186/s12870-026-09546-y

**Published:** 2026-07-15

**Authors:** Xiaotian Liao, Xu Zhang, Minhong Zhang, Yu Li, Ning’ao Wang, Hongzhi He

**Affiliations:** 1 Guangdong Engineering Technology Research Centre of Modern Eco-agriculture and Circular Agriculture, Guangzhou, China; 2 Key Laboratory of Agro-Environment in the Tropics, Ministry of Agriculture of the People’s Republic of China, Guangzhou, China; 3 College of Natural Resources and Environment, South China Agricultural University, Guangzhou, 510642 China

**Keywords:** Rice lodging, Cyanobacterial extract, Biofertilizer, Biostimulant

## Abstract

**Background:**

Lodging is a major threat to rice production, underscoring the need for sustainable technologies to improve lodging resistance. This study evaluated the potential of cell extracts from several microalgae and cyanobacteria to induce lodging resistance in rice.

**Results:**

Pot experiments identified the extract from *Nostoc* sp. SCAU-13 as effective, significantly increasing stem diameter, stem wall thickness, and bending resistance, while reducing the lodging index. Optimization of the extraction method revealed that the aqueous extract (AQE) was the most effective, decreasing the lodging index by 25.2%. Metabolomic analysis showed that AQE is rich in 967 metabolites, notably plant hormones, flavonoids, and lipids. Transcriptomic analysis indicated that AQE treatment significantly altered gene expression in rice stems. Differentially expressed genes were significantly enriched in pathways such as ribosome biogenesis, phenylpropanoid biosynthesis, linoleic acid metabolism, and glutathione metabolism. The resistance mechanism involves remodeling the gene expression network: downregulating defense-related genes to attenuate the “defense alert”; upregulating ribosome synthesis genes and growth regulators to promote protein synthesis and stem reinforcement; and modulating the phenylpropanoid pathway to enhance cell wall lignification. Field trials demonstrated that applying AQE alone reduced the lodging index by 15%. Furthermore, combining AQE with *Constrictifilum* sp. SCAU-26 increased rice yield even under a 20% reduction in chemical nitrogen fertilizer.

**Conclusion:**

This study employed multi-omics approaches to elucidate the molecular mechanism by which *Nostoc* sp. SCAU-13 extract enhances lodging resistance in rice, laying a foundation for the development of green biostimulants derived from cyanobacteria.

**Supplementary Information:**

The online version contains supplementary material available at https://doi.org/10.1186/s12870-026-09546-y.

## Introduction

Rice (*Oryza sativa* L.) is one of the most widely cultivated staple crops and is vital to global food security. Modern breeding programs that prioritize high biomass have inadvertently increased the mechanical load on basal stem tissues. This, combined with excessive nitrogen fertilization in intensive cultivation systems, has made lodging a serious threat to rice production. Severe lodging impairs the synthesis, translocation, and storage of photosynthates, resulting in reduced grain yield and quality and complicating mechanical harvesting [11, 18]. Lodging is influenced not only by adverse weather and suboptimal management but also by the plant’s inherent physical and chemical properties and their underlying molecular mechanisms [11, 18, 30, 44]. Major factors affecting lodging resistance include plant height, panicle type, stem morphology, and chemical composition [30, 44]. Although the use of lodging-resistant varieties and plant growth regulators (PGRs) is an effective strategy to reduce lodging risk, resistance breeding is time-consuming and genetically unstable, and synthetic PGRs may pose environmental risks [18].

Lodging is closely related to plant morphological traits and stem physical strength [28]. Increased plant height and a higher center of gravity elevate the lodging risk. As a result, there is a trade-off between maximizing rice yield and minimizing lodging: high grain yield often requires a taller plant architecture with a higher center of gravity, which may increase lodging susceptibility [18, 45]. Conversely, reducing plant height to lower lodging risk may limit yield potential, as an optimal height is essential for canopy photosynthesis and biomass accumulation [46]. Thus, sophisticated strategies are needed to simultaneously achieve high yield and low lodging risk in paddy fields [7, 10].

Microalgae and cyanobacteria are photosynthetic microorganisms found in diverse environments; they exhibit rapid growth, high photosynthetic efficiency, and produce a wide array of metabolites. They can improve soil fertility through nitrogen fixation, phosphate solubilization, and potassium release, and secrete phytohormone-like substances that stimulate root development and tillering. Moreover, they may play important roles in enhancing crop tolerance to both biotic and abiotic stresses [6]. Accordingly, microalgae and cyanobacteria can serve as biofertilizers, biopesticides, biostimulants, and stress resistance modulators in agriculture [12, 34, 35], offering a sustainable approach to increase crop productivity while reducing environmental pollution [8]. However, although microalgae and cyanobacteria are generally known to promote rice growth and yield, they may simultaneously increase lodging risk. For example, our previous work showed that the strain SCAU-26, despite its strong yield-promoting ability [34], increased lodging susceptibility. Reports on the use of microalgal or cyanobacterial extracts specifically to enhance rice lodging resistance are rare. Given the structural and bioactive diversity of secondary metabolites produced by microalgae and cyanobacteria, we hypothesized that specific algal extracts could enhance stem mechanical strength and reduce the lodging index.

Therefore, this study aimed to: (1) screen microalgal and cyanobacterial extracts for their ability to enhance rice lodging resistance through pot experiments; (2) optimize the extraction method for the active compounds; (3) investigate the mechanisms by which the microalgal/cyanobacterial extract induces lodging resistance in rice; and (4) validate the lodging resistance effect in field plot experiments.

## Materials and methods

### Experimental materials

#### Rice cultivars

The rice (*Oryza sativa* L.) used in the pot experiment was the thermo-sensitive inbred cultivar Huanghuazhan (provided by the Rice Research Institute, Guangdong Academy of Agricultural Sciences). The cultivar used in the field application experiment was Zengkexinxuan Simiao 2 (provided by the Zengcheng Agricultural Science Institute, Guangzhou).

#### Experimental soil

The soil used for the pot experiment was paddy soil collected from the Yuejinbei Farm of South China Agricultural University. Its basic physicochemical properties were as follows: pH 6.7, alkaline hydrolyzable nitrogen 138 mg/kg, available phosphorus 53.9 mg/kg, organic matter 22.6 g/kg, and available potassium 243 mg/kg.

#### Microalgal/cyanobacterial strains

Four microalgal/cyanobacterial strains were used: *Chlorella* sp. HS02 (HS; NCBI accession: PQ824500), *Chlorella* sp. HH01 (HH; NCBI accession: PQ357523), *Constrictifilum* (previously known as *Anabaena* or *Trichormus*) sp. SCAU-26 (TS; NCBI accession: PX856690), and *Nostoc* sp. SCAU13 (J; NCBI accession: OR874847). All strains were isolated, purified, and identified in our laboratory and cultured in BG11 medium.

#### Reagents

Chemicals and reagents used included 30% hydrogen peroxide, 5% sodium hypochlorite, sulfuric acid, 95% ethanol, perchloric acid, nitric acid, and benzyl alcohol. A plant soluble sugar content assay kit was purchased from the Nanjing Jiancheng Bioengineering Institute. Unless otherwise specified, all reagents were of analytical grade.

### Experimental design

#### Pot screening experiment for microalgae and cyanobacteria extracts inducing rice lodging resistance

The pot experiment was conducted from March to July 2023 at the Ecological Farm of South China Agricultural University. Five treatments were established, all received 10 g of identical 15:15:15 NPK basal fertilizer: a conventional fertilization control (CK) and treatments with extracts from the four different microalgae/cyanobacteria (HS, HH, TS, and J). Each treatment had four replicates, and pots were randomly arranged to minimize systematic errors due to light, temperature, and humidity gradients.

Preparation of the four microalgal/cyanobacterial extracts: Under sterile conditions, 800 mL of BG11 liquid medium in a 2 L Erlenmeyer flask was inoculated with 40 mL of algal/cyanobacterial culture and incubated at 28 °C under 3000 lx with a 16 h:8 h light/dark cycle. Flasks were shaken twice daily to ensure uniform growth. For extract preparation, J and TS were harvested on day 12; HH and HS on day 8. Cells were collected by centrifugation, washed three times with ice-cold PBS (pH 7.0) to remove residual medium, and lyophilized. The dried cell pellets were ground to a fine powder in a pre-chilled mortar with liquid nitrogen and quartz sand for 10 min; complete cell disruption was confirmed microscopically. Distilled water was added at a ratio of 1:20 (w/v, dry weight to water), and the mixture was subjected to ice-bath ultrasonication (20 kHz, 200 W, 30 s on/30 s off) for 30 min. The extract was filtered through filter paper and diluted with distilled water to five times the original mixture volume for foliar spraying. Each pot received 50 mL of extract solution, applied until droplets appeared at leaf margins and the stem base, indicating thorough wetting without runoff.

Pots (34 cm diameter × 30 cm height) were each filled with 8.5 kg of soil and 1.0 kg of fine river sand, thoroughly mixed. Each treatment received an extract amount equivalent to 2 g of microalgae/cyanobacteria dry weight. Extracts were applied three times by foliar spraying: at the tillering stage (15–20 days after transplanting), booting stage (5–7 days before panicle emergence), and grain-filling stage (5–7 days after heading). At maturity, samples were collected to measure primary indicators (lodging index and stem mechanical strength) and secondary indicators (yield and stem chemical composition).

#### Pot experiment for optimization of algal/cyanobacterial extraction methods

Four treatments were established, all receiving the same NPK compound basal fertilizer: conventional fertilization control (CK), hot water extract (HWE), liquid nitrogen grinding with ultrasonication-assisted aqueous extract (AQE), and alcohol extract (ALE). Each treatment had four replicates. Pot specifications and soil preparation were as described in Sect.  2.2.1, and pots were randomly arranged to minimize systematic errors from light, temperature, and humidity gradients.

Preparation of the three extracts: BG11 medium without sodium carbonate was prepared and inoculated with 5% (v/v) of the respective culture in the logarithmic growth phase, then incubated at 28 °C under 5000 lx with a 16 h:8 h light/dark cycle. Cells were harvested on day 12 by centrifugation and freeze-dried into powder. The three extraction methods were as follows: (1) HWE: 1 g of dry powder was shaken with 10 mL distilled water for 3 min, extracted in a 98 °C water bath for 5 h, and then filtered or centrifuged. The residue was re-extracted 1–2 times using the same procedure. The combined filtrates/supernatants were brought to 50 mL. (2) AQE: 1 g of powder was ground thoroughly for 10 min in a pre-cooled mortar with liquid nitrogen and quartz sand, after which 10 mL distilled water was added and shaken for 3 min. The suspension was subjected to ultrasonic cell disruption (500 W, 40 min per cycle, 5 cycles) in an ice bath until complete cell breakage was confirmed microscopically. The mixture was centrifuged or vacuum-filtered, and the residue was re-extracted 1–2 more times. The combined extracts were brought to 50 mL. (3) ALE: 1 g of dry powder was placed in a 100 mL glass Erlenmeyer flask, 10 mL ethanol was added and shaken for 3 min, then extracted with stirring in an 80 °C water bath for 2 h. After vacuum filtration, the residue was re-extracted twice following the same steps. The combined filtrates were brought to 50 mL with distilled water. All extracts were filtered through filter paper and stored at 4 °C in the dark for no more than 72 h. Applications were performed three times (tillering, booting, and grain-filling stages) by foliar spraying. At maturity, stem strength, internode morphological characteristics, lodging resistance, and yield-related traits were measured.

#### Metabolite analysis

After expansion cultivation of the optimal strain for 12 days, cells were collected by centrifugation and freeze-dried to prepare the aqueous extract for metabolomic analysis. The cultivation and aqueous extraction procedures were the same as in Sect.  2.2.2. A 10 mL aliquot of the extract was centrifuged (4 °C, 1000 g, 5 min); the supernatant was discarded, and the cell pellet (~ 50 µL) was transferred to a 2 mL sample tube, flash-frozen in liquid nitrogen for 15 min, stored at − 80 °C, and sent for analysis.

#### Transcriptomics experiment

Two treatments were established: conventional fertilization control (CK) and AQE treatment, each with three replicates. AQE-treated rice received the aqueous extract twice, at the tillering and jointing stages. Seventy-two hours after the jointing-stage application, fresh stem tissues were sampled. Surface dust was removed with sterile water, and samples were immediately placed into pre-cooled cryotubes, wrapped in foil, flash-frozen in liquid nitrogen for 30 min, stored at − 80 °C, and shipped on dry ice for analysis.

#### Field application experiment on rice

The field experiment was conducted on a farm in Guangzhou. Three treatments were established: CK, AQE, and AQE + TS, each with three replicate plots. The nine plots were randomly distributed, each covering approximately 200 m², and separated by ridges approximately 2 m wide. The soil was a clay loam with uniform fertility. Treatment details were as follows: CK (conventional fertilization: NPK compound fertilizer (15-15-15) applied once at the seedling stage at 750 kg/ha); AQE (conventional fertilization plus AQE foliar spray applied twice, at the tillering and booting stages, at 5 L per plot each time); AQE + TS (20% reduction in nitrogen fertilizer + AQE as above + live cyanobacterial strain TS cultured to the logarithmic growth phase, also applied at 5 L per plot). Water management followed standard practices: shallow water for transplanting, appropriate drying at tillering, deep water at booting, and shallow water with alternating wetting and drying at grain filling. During the experiment, weather was predominantly cloudy or overcast, with moderate rainfall and average daily temperatures of 20–26 °C. At maturity, rice samples were collected from three randomly selected 1 m² areas per plot to measure primary indicators (lodging index and stem mechanical strength) and secondary indicators (yield and stem chemical composition).

### Measurements and methods

#### Mechanical strength indices of rice stems

Approximately 20 days after heading, 10 uniform main stems were randomly selected from each treatment. Plant height, internode length, and height of the center of gravity were measured. The breaking resistance (F, kg) of the basal second internode was determined using a three-point bending test: the internode was placed on two supports 5 cm apart, and a plant stem strength tester (YYD-1) was applied downward at the midpoint until the stem broke; the force at failure was recorded. Stem diameter and stem wall thickness of the same internode were measured with a vernier caliper. Lodging-related mechanical parameters were calculated [22] as follows:

1. Bending moment (WP, g·cm) = SL × FW, where SL (cm) is the length from the breaking point to the panicle tip, and FW (g) is the fresh weight of the same segment.
2. Breaking moment (M, g·cm) = L × F / 4 × 10³, where F (kg) is the breaking force and L (cm) is the distance between supports (5 cm).
3. Lodging index (LI) = WP / M.

The field lodging score was calculated as the product of the percentage of lodged area and the angle severity index: field lodging score = (lodged area %) × (angle severity).

#### Rice chemical indicators

Non-structural carbohydrate (NSC) and structural carbohydrate (cellulose and lignin) contents were measured. NSC content was determined according to Yoshida [36]: (1) Accurately weigh 0.05 g of stem powder (passed through an 80-mesh sieve), add 2 mL water, and heat in a boiling water bath for 30 min. (2) Add 2 mL of 9.2 mol/L perchloric acid, react for 10–20 min, centrifuge at 5000 rpm, discard the residue, and collect the supernatant in a 50 mL volumetric flask. Repeat step (2) three times. NSC content was quantified by the anthrone colorimetric method at 620 nm. Cellulose content was determined following Updegraff [37]: Accurately weigh 0.05 g of sieved powder, add 5 mL acetic/nitric acid reagent, and heat in a boiling water bath for 30 min. After cooling to room temperature, centrifuge at 5000 rpm for 15 min, discard the supernatant, and retain the residue. Repeat the above steps three times. Then add 5 mL of 72% sulfuric acid, let stand at room temperature overnight until the residue is completely dissolved. Measure cellulose content by the anthrone colorimetric method at 620 nm. Lignin content was determined as described by Ishimaru et al. [13] and Zhang et al. [43]: Accurately weigh 1 g of sieved sample, extract with benzene–alcohol for 4 h, and air-dry. Add 15 mL of 72% sulfuric acid and incubate in an 18–20 °C water bath with constant shaking for 2.5 h. Transfer the sample to a 500 mL conical flask, dilute with distilled water to 3% sulfuric acid, and boil for 4 h. Allow the acid-insoluble lignin to precipitate, then dry at 105 °C to constant weight. Lignin content (%) = W / S × 100, where W is the weight of acid-insoluble lignin and S is the sample weight.

#### Extract composition analysis

UHPLC-MS/MS analysis was performed on a Novogene UHPLC system coupled to a Q Exactive™ HF mass spectrometer using a Hypersil Gold column (100 × 2.1 mm, 1.9 μm). Raw data files were processed with CD 3.3 library search software. Metabolites were identified by screening retention time, mass-to-charge ratio, and other parameters. After correction, quantitative integration, database matching, background ion removal, and normalization of raw quantitative results, metabolite identification and relative quantification were obtained.

#### Rice transcriptomics analysis

Two treatment groups, CK and AQE, were established with three biological replicates each. Total RNA was extracted using the Tiangen RNA isolation kit (Catalog No. DP762-T1A). Transcriptome libraries were sequenced on the Illumina NovaSeq 6000 platform in paired-end mode (PE150, read length 150 bp), yielding approximately 6 Gb of raw data per sample. Raw reads were filtered with Fastp (v0.22.0) to remove adapter sequences and reads with an average quality score below Q20, resulting in high-quality clean reads. The rice reference genome and annotation files were downloaded from a public database. HISAT2 (v2.1.0) was used to build the genome index and align clean reads to the reference genome. Read counts were quantified with HTSeq (v0.9.1), and gene expression levels were normalized as fragments per kilobase of transcript per million mapped reads (FPKM). Differential expression analysis was performed using DESeq2 (v1.38.3), with significantly differentially expressed genes (DEGs) defined by |log2(fold change)| > 1 and adjusted p-value < 0.05. Subsequent analyses included hierarchical clustering heatmaps, volcano plots, Venn diagrams, GO and KEGG enrichment analyses, and transcription factor prediction.

For transcriptomic data validation, qRT-PCR was performed on seven candidate genes that showed significant differential expression and have relatively well-defined functional annotations in rice (Table S1). Total RNA was extracted from the same samples using the same protocol as for RNA-seq. RNA integrity was checked by agarose gel electrophoresis, and qualified RNA was reverse-transcribed into cDNA using the Advantage^®^ RT-for-PCR Kit (Takara). Primer sequences, amplicon sequences, and gene annotations are listed in Table S1; Actin was used as the internal reference. PCR amplification was conducted with AceQ qPCR SYBR Green Master Mix (Vazyme) on a LightCycler^®^ 480II 384-well real-time PCR system (Roche) under the following conditions: 95 °C for 5 min, followed by 40 cycles of 95 °C for 15 s and 60 °C for 30 s.

#### Rice yield measurement

Total yield per pot was recorded. The number of effective panicles per hill (panicles with ≥ 5 filled grains), grains per panicle, unfilled grains per panicle, 1000-grain weight, and total fresh and dry weights per pot were determined by conventional methods. Seed-setting rate was calculated. For the field experiment, rice from a 1 m² area was harvested, threshed, and weighed fresh, then dried to constant weight to calculate yield.

#### Rice quality analysis

Brown rice rate was determined using a huller, and milled rice rate was obtained after milling. Moisture, amylose, and protein contents were measured using a grain analyzer.

### Data processing

Experimental data were organized using Excel 2019. Statistical analyses were performed with SPSS 26: independent-samples *t*-test for pairwise comparisons and one-way ANOVA followed by Duncan’s multiple range test for multiple comparisons. Significance was set at *p* < 0.05. Graphs were generated with Origin 2024 and Excel 2019.

## Results and analysis

### Pot experiment on the induction of rice lodging resistance by different algal/cyanobacterial extracts

**Fig. 1:**
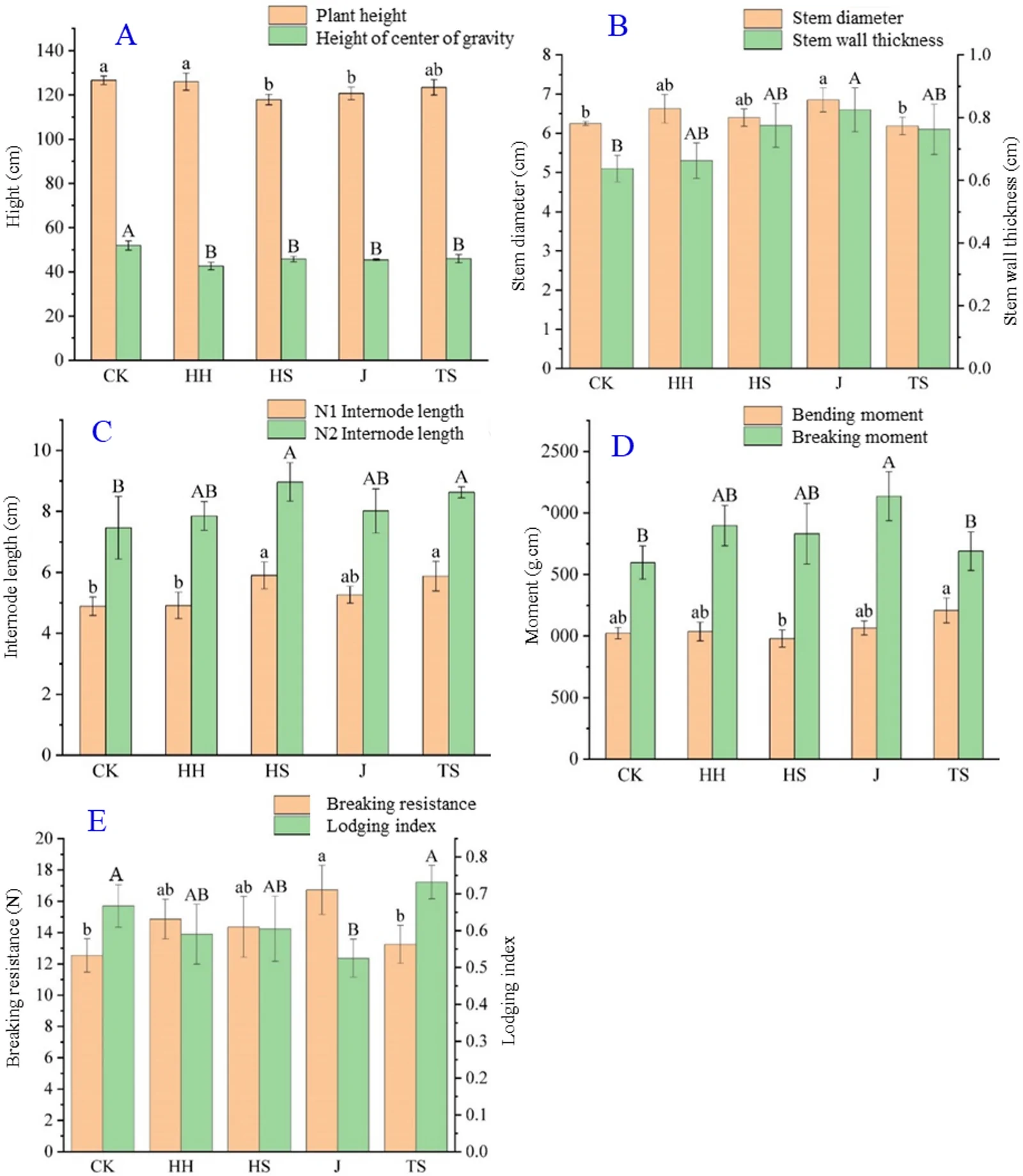
Screening of different algal/cyanobacterial extracts for inducing rice lodging resistance in a pot experiment. **A** Plant height and height of center of gravity; (**B**) Stem diameter and wall thickness; (**C**) Internode length; (**D**) Bending moment and breaking moment; (**E**) Breaking resistance and lodging index. Error bars represent standard deviation. Different letters above bars indicate statistically significant differences among treatments (*p* < 0.05). The same notation applies below

The effects of extracts from four different microalgae and cyanobacteria (HH, HS, J, and TS) on rice stem height, center of gravity height, stem diameter, internode length, breaking resistance, and lodging index are shown in Fig. 1. As shown in Fig. 1A, plant height was significantly lower under HS and J treatments than CK (by 6.9% and 4.7%, respectively), but did not differ significantly from TS and HH treatments. Center of gravity height was significantly reduced by all four extracts compared to CK, with decreases of 17.9% (HH), 11.9% (HS), 12.5% (J), and 11.6% (TS); no significant differences were observed among the four extract treatments. Stem diameter was greatest under J treatment, being significantly larger than CK and TS by 8.7% and 9.6%, respectively, whereas HH, HS, and TS did not differ significantly from CK. Stem wall thickness was also highest under J treatment, with a significant 22.6% increase over CK, while the other treatments were similar to CK (Fig. 1B). For the lengths of the first and second basal internodes, HS and TS were significantly greater than CK, whereas HH and J did not differ from CK (Fig. 1C). The bending moment under TS was significantly higher than under HS (by 18.9%), but did not differ significantly from CK, HH, or J. The breaking moment under J was significantly higher than CK (by 25.2%) and significantly higher than TS, but similar to HH and HS (Fig. 1D). Mean breaking resistance was highest under J, significantly exceeding CK and TS by 25% and 20.8%, respectively, and not significantly different from HH and HS. Correspondingly, the lodging index was lowest under J treatment, 21.2% lower than CK (Fig. 1E). These results indicate that the J extract has strong potential for enhancing rice lodging resistance, prompting further optimization of the extraction method.

### Pot experiment on optimization of extraction methods

**Fig. 2:**
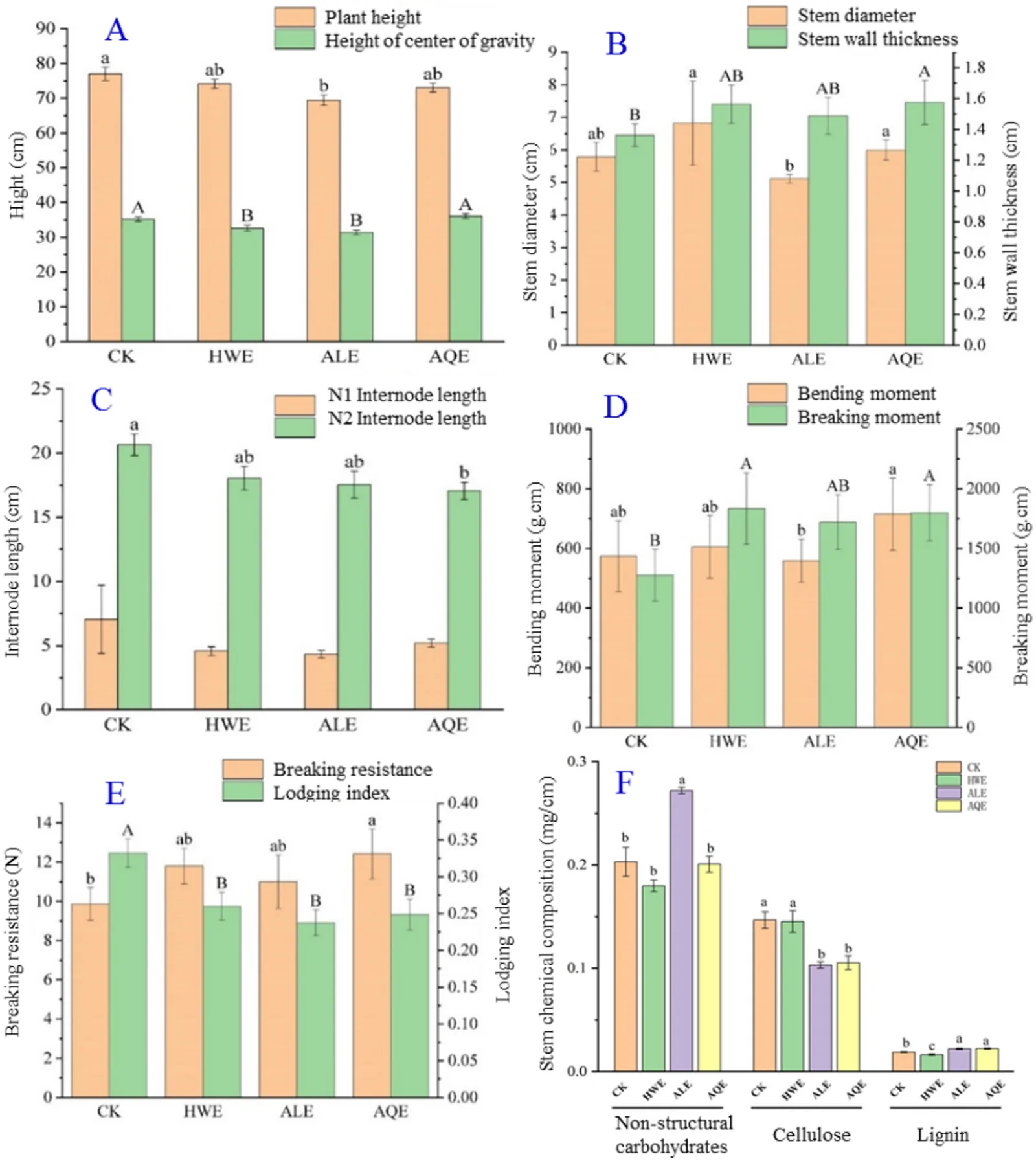
Effects of different extraction methods on rice growth, morphology, lodging resistance, and stem chemical composition. **A** Plant height and height of center of gravity; (**B**) Stem diameter and wall thickness; (**C**) Internode length; (**D**) Bending moment and breaking moment; (**E**) Breaking resistance and lodging index; (**F**) Stem chemical composition

The effects of different extraction methods on rice growth, stem morphology, and chemical composition are shown in Fig. S1 and Fig. 2. As shown in Fig. 2A, only ALE significantly reduced plant height, by 9.8% relative to CK. Plant heights under HWE and AQE were numerically lower than CK but not significantly different. Center of gravity height under AQE was not significantly different from CK, and both were significantly higher than those under HWE and ALE. Stem diameter did not differ significantly from CK under any treatment, although HWE and AQE were significantly greater than ALE. Stem wall thickness was highest under AQE, 13.5% greater than CK, but not significantly different from HWE and ALE (Fig. 2B). The first internode length was greatest in CK, with no significant differences among treatments. The second internode length was also greatest in CK; AQE significantly shortened it by 17.4% compared to CK, while HWE and ALE did not differ significantly from AQE (Fig. 2C). The bending moment was highest under AQE, significantly higher than ALE, but not significantly different from CK. The breaking moment under HWE and AQE was significantly higher than CK, with AQE showing a 29.1% increase, and neither differed significantly from ALE (Fig. 2D). Breaking resistance under AQE was significantly higher than CK by 20.5%, but not significantly different from HWE or ALE. Lodging indices of all extract treatments were significantly lower than CK, with HWE, ALE, and AQE reducing the index by 21.7%, 28.5%, and 25.2%, respectively, and no significant differences among the three extraction methods (Fig. 2E). As shown in Fig. 2F, NSC content under ALE was significantly higher than CK and the other treatments, whereas the other treatments did not differ from CK. Stem cellulose content under CK and HWE was significantly higher than under ALE and AQE, which did not differ from each other. Stem lignin content under ALE and AQE was significantly higher than CK (by 13.7% and 14.9%, respectively), while HWE had the lowest lignin content, significantly lower than CK.

The effects of different extraction methods on rice yield components are summarized in Table 1. The number of effective panicles was highest under CK, with no significant differences among treatments. Grain number per panicle was highest under HWE, followed by ALE, and lowest under CK; HWE and ALE significantly increased grain number by 6.5% and 5.2% compared to CK, respectively, but did not differ significantly from AQE. The number of unfilled grains was lowest under ALE, 26.8% lower than CK. Filled grain numbers under ALE and HWE were significantly higher than CK (by 7.1% and 6.8%, respectively), while AQE did not differ significantly from them. Thousand-grain weight was highest under AQE, significantly exceeding CK, HWE, and ALE by 21.9%, 16.4%, and 12.8%, respectively; ALE was also significantly higher than CK by 10.5%, but not significantly different from HWE. Seed-setting rate was highest under ALE (95%), with no significant differences among treatments. Aboveground fresh weight was highest under AQE, 15.6% higher than CK, but not significantly different from HWE and ALE. Grain yield under all extract treatments was significantly higher than CK, with AQE yielding the highest and significantly greater than HWE and ALE. AQE, HWE, and ALE increased yield by 25.9%, 16.4%, and 20%, respectively, relative to CK. Based on these comprehensive results, AQE was selected as the optimal extraction method.

**Table 1:** Effects of different extraction methods on rice yield in potted experiments

Treatment	Effective number of panicles	Number of grains per panicle	Number of empty grains	Number of filled grains	Thousand grain weight (g)	Seed setting rate (%)	Total yield (g/pot)
CK	11.4 ± 1.4a	163 ± 33b	10.3 ± 5.0a	153 ± 31b	17.6 ± 0.5c	93.6 ± 2.8a	35.3 ± 0.7c
HWE	11.3 ± 0.9a	174 ± 9a	10.7 ± 3.6a	164 ± 8a	18.8 ± 0.5bc	93.9 ± 2.0a	42.2 ± 1.1b
ALE	10.4 ± 1.4a	172 ± 35a	7.56 ± 3.4b	164 ± 34a	19.6 ± 0.6b	95.5 ± 1.9a	44.1 ± 0.6b
AQE	10.1 ± 0.9a	169 ± 33ab	11.2 ± 5.5a	158 ± 32ab	22.5 ± 0.9a	93.3 ± 3.3a	47.6 ± 1.8a

different letters in the same column indicate significant differences (*p* < 0.05)

### Chemical composition analysis of the extract

Figure S2 shows the major metabolite classes identified in AQE. In positive ion mode, 610 metabolites were detected (Table S2). Lipids and lipid-like molecules were the most abundant (28.73%), followed by organoheterocyclic compounds (18.33%), organic acids and derivatives (18.1%), and benzenoids (11.54%). Nucleosides, nucleotides, and analogues; organic oxygen compounds; phenylpropanoids and polyketides; alkaloids and derivatives; and organic nitrogen compounds each accounted for less than 10% (Fig. S2A). In negative ion mode, 357 metabolites were detected (Table S2). Lipids and lipid-like molecules were again predominant (38.78%), followed by organic acids and derivatives (16.33%) and nucleosides, nucleotides, and analogues (10.54%). The remaining classes each comprised less than 10% (Fig. S2B). Table 2 lists substances potentially related to growth promotion and lodging resistance, including fatty acids and lipids, organic acids and derivatives, nucleotides/nucleosides, flavonoids, lignin and lignans, among others.

**Table 2:** Potential candidate substances in cyanobacterial extracts for inducing lodging resistance in rice

Name	Relative percentage content (%)	Formula	Molecular weight	RT [min]	m/z	Class_I	Class_II
DL-Arginine	2.05	C6H14N4O2	174.1117	1.335	175.119	organic acids and derivatives	carboxylic acids and derivatives
N-Acetylglucosamine	0.196	C8H15NO6	221.0898	1.431	256.0594	organic oxygen compounds	organooxygen compounds
Proline	6.53	C5H9NO2	115.0636	1.448	116.0709	organic acids and derivatives	carboxylic acids and derivatives
Citric acid	0.746	C6H8O7	192.0264	1.572	191.0191	organic acids and derivatives	carboxylic acids and derivatives
N-Acetylaspartic acid	0.185	C6H9NO5	175.0472	2.017	174.0399	organic acids and derivatives	carboxylic acids and derivatives
Lipoic acid	14.3	C8H14O2S2	206.0424	2.698	205.0351	organoheterocyclic compounds	dithiolanes
Adenosine	1.96	C10H13N5O4	267.0966	3.136	268.1039	nucleosides, nucleotides, and analogues	purine nucleosides
2’-Deoxyadenosine	3	C10H13N5O3	251.1016	3.537	252.1089	nucleosides, nucleotides, and analogues	purine nucleosides
3-Hydroxyphenylacetic acid	0.228	C8H8O3	152.0465	4.212	153.0538	benzenoids	phenols
2’-Deoxyguanosine	2.09	C10H13N5O4	267.0966	4.221	268.1038	nucleosides, nucleotides, and analogues	purine nucleosides
Methionine	0.188	C5H11NO2S	149.0512	4.51	150.0584	organic acids and derivatives	carboxylic acids and derivatives
L-Phenylalanine	23.17	C9H11NO2	165.0791	4.975	166.0863	organic acids and derivatives	carboxylic acids and derivatives
Pantothenic acid	0.153	C9H17NO5	219.1104	5.115	218.1031	organic oxygen compounds	organooxygen compounds
Indole	0.147	C8H7N	117.0581	5.281	118.0654	organoheterocyclic compounds	indoles and derivatives
Abscisic Acid	0.0003	C15H20O4	264.136	5.665	265.1433	lipids and lipid-like molecules	prenol lipids
Indole-3-acetic acid	0.087	C10H9NO2	175.0634	5.767	176.0707	organoheterocyclic compounds	indoles and derivatives
Olivetol	0.241	C11H16O2	180.1149	6.187	181.1222	benzenoids	phenols
Dihydrojasmone	0.151	C11H18O	166.1357	6.3	149.1324	organic oxygen compounds	organooxygen compounds
Corchorifatty acid F	9.89	C18H32O5	328.2252	6.316	327.2179	lipids and lipid-like molecules	fatty acyls
Prostaglandin K2	0.196	C20H30O5	332.1959	6.513	333.2032	lipids and lipid-like molecules	fatty acyls
Prohydrojasmon	0.141	C15H26O3	254.1882	6.947	253.1809	lipids and lipid-like molecules	fatty acyls
Oleamide	3.38	C18H35NO	281.2716	9.811	304.2607	lipids and lipid-like molecules	fatty acyls

### Rice transcriptomic analysis

**Fig. 3:**
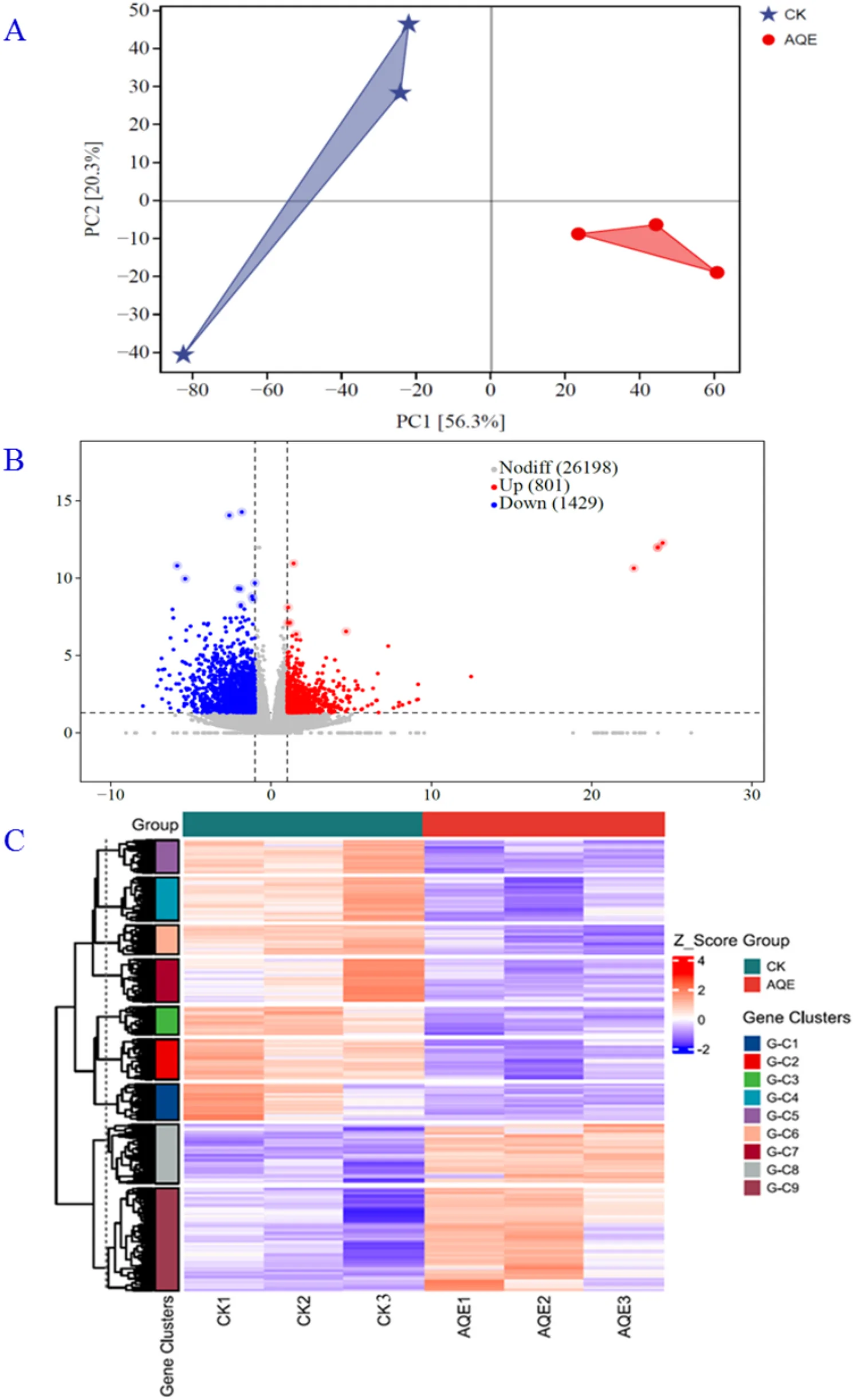
Transcriptomic analysis of differentially expressed genes. **A** PCA score plot; (**B**) Volcano plot; (**C**) Heatmap

**Fig. 4:**
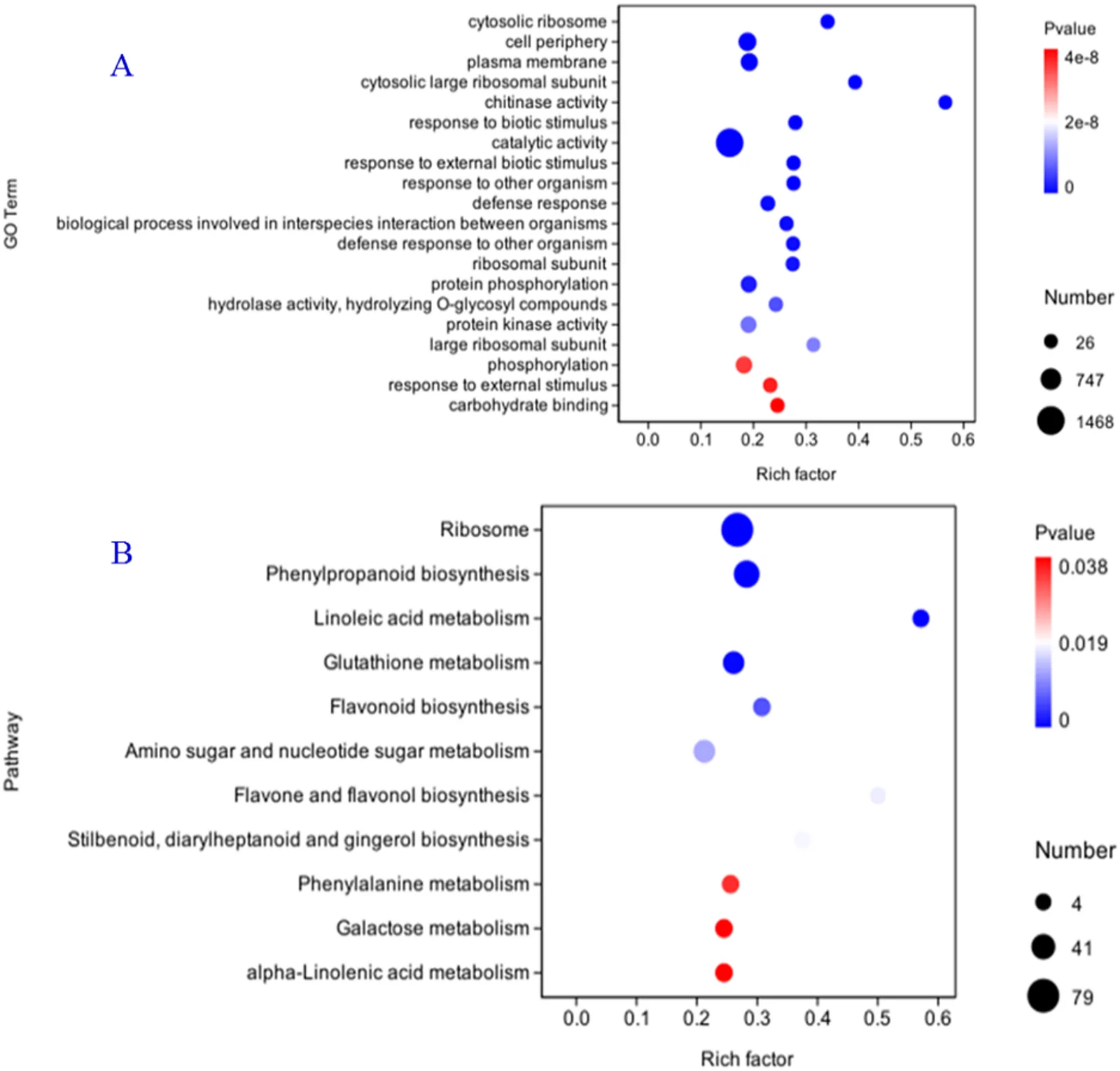
Enrichment analysis of DEGs. **A** GO enrichment; (**B**) KEGG enrichment

The transcriptomic results are presented in Figs. 3 and 4, Fig. S3, and Tables S3–S8. PCA of the samples (Fig. 3A) showed a clear separation between CK and AQE, indicating distinct gene expression profiles. DEGs were identified with |log_2_(fold change)| > 1 and *p* < 0.05. Comparison between AQE and CK revealed 2230 DEGs, of which 801 were upregulated and 1429 downregulated (Fig. 3B, Table S4).

Hierarchical clustering of DEGs (Fig. 3C) revealed distinct expression patterns between treatments, demonstrating that AQE induces substantial changes in stem-related genes. Compared to CK, AQE significantly increased the expression of genes potentially associated with lodging resistance, including *AP2*, *OsARF13*, *OsbZIP25*, *GRF1*, *OsNAC5*, *OsIDD3*/*4*/*5*, and *YAB3/5*. In contrast, genes such as *BZIP23*, *ERF3*, *ONAC038*, *OsERF_049*, *OsISC12*, *OsMFT1*, *OsMTP11*, *OsOAT*, *OsTBL34*, *OsWRKY30*, *OsY37*, and *OsZNL* was significantly downregulated.

GO enrichment analysis of DEGs (AQE vs. CK) is shown in Fig. 4A. Significantly enriched (*p* < 0.01 and *FDR* < 0.01) biological process terms included response to biotic stimulus, response to external biotic stimulus, response to other organism, defense response, biological process involved in interspecies interaction between organisms, defense response to other organism, protein phosphorylation, phosphorylation, response to external stimulus, among 38 terms. Except for “microtubule-based process”, in which most genes were upregulated, all other terms contained more downregulated than upregulated genes. The majority of genes related to defense or response to external stimuli were significantly downregulated; for instance, “response to external biotic stimulus” included 12 upregulated and 72 downregulated genes, and “defense response” included 26 upregulated and 112 downregulated genes. These results indicate that AQE treatment broadly downregulated defense- and stimulus-responsive genes in rice, reflecting a systemic modulation of the rice immune system by cyanobacterial bioactive compounds. This coordinated downregulation of defense-related genes suggests an “immune silencing–resource optimization–growth promotion” strategy, enabling rice to reduce unnecessary defense expenditure under non-stressful conditions and redirect resources toward growth, development, and basal stress tolerance.

Significantly enriched cellular component terms included cytosolic ribosome, cell periphery, plasma membrane, cytosolic large ribosomal subunit, ribosomal subunit, large ribosomal subunit, anchored component of membrane, anchored component of plasma membrane, ribosome, and others—16 terms in total. With the exception of cell periphery, plasma membrane, and intrinsic component of plasma membrane, where most genes were downregulated, all other terms were dominated by upregulated genes. This pattern suggests a bidirectional regulation of cellular components by cyanobacterial bioactive substances: activation of ribosome-related genes to enhance protein synthesis, supporting growth and photosynthesis, alongside suppression of certain membrane protein genes, potentially optimizing resource allocation and improving stress resilience.

Significantly enriched molecular function terms included chitinase activity, catalytic activity, hydrolase activity (hydrolyzing O-glycosyl compounds), protein kinase activity, carbohydrate binding, hydrolase activity (acting on glycosyl bonds), oxidoreductase activity, kinase activity, among 30 terms. With the exception of structural constituent of ribosome, structural molecule activity, microtubule binding, receptor serine/threonine kinase binding, and microtubule motor activity—where most genes were upregulated—all other terms contained more downregulated than upregulated genes. This indicates a differentiation in molecular functions: structural functions (ribosome, microtubules) were upregulated, while catalytic functions (hydrolases, kinases) were largely downregulated. Together, these findings suggest that the cyanobacterial extract alleviates the “defense-alert” state in rice. By coordinating cellular metabolism and structural remodeling, resources are directed toward growth and development, which aligns with the “symbiotic adaptability” of cyanobacteria as biostimulants—mimicking environmental safety signals through bioactive substances (e.g., polysaccharides, hormones) to steer rice into a “growth-priority, energy-efficient” mode.

KEGG enrichment analysis (Fig. 4B) showed that DEGs were significantly enriched (*p* < 0.05 and *FDR* < 0.05) in the ribosome (79 genes), phenylpropanoid biosynthesis (50 genes), linoleic acid metabolism (8 genes), and glutathione metabolism (30 genes) pathways. All 79 DEGs in the ribosome pathway were upregulated (Fig. S3A), suggesting that AQE enhances protein synthesis capacity, consistent with the observed increase in protein content. In the phenylpropanoid biosynthesis pathway, *Os08g0543400* encoding shikimate O-hydroxycinnamoyltransferase (HCT) and several peroxidase genes (*PRX12/24/58/87/134*), were significantly upregulated, whereas many other *PRX* and *POX* genes were downregulated (Fig. S3B). HCT in rice catalyzes the formation of esters such as caffeoyl shikimate from shikimate and hydroxycinnamoyl-CoAs, which are precursors for phenolic compounds (e.g., ferulic acid, cinnamic acid) involved in disease resistance and cell wall lignification. Concurrently, classic flavonoid biosynthesis enzymes (trans-cinnamate 4-monooxygenase, caffeoyl-CoA O-methyltransferase, chalcone isomerase, chalcone synthase, flavonoid 3’-monooxygenase, phlorizin synthase, anthocyanidin reductase) were downregulated. Because HCT competes for the common substrate 4-coumaroyl-CoA, its upregulation likely diverts carbon flux away from flavonoid/anthocyanin synthesis toward lignin precursors. In linoleic acid metabolism, three linoleate 9 S-lipoxygenase genes (*LOX-L2*, *OsLOX1*, and *Osr9-LOX1*) were drastically downregulated (by 95%, 54%, and 65%, respectively). Within the lipoxygenase gene family, only *LOX2:Hv:3* was significantly upregulated (2.93-fold), while *SPH*, *OsLOX8/11*, and *Lox2:Os:1* were downregulated by 55%–92% (Fig. S3C). This indicates differential regulation within the *LOX* gene family: defense-related *LOXs* were suppressed, whereas a development-associated *LOX* was activated, suggesting a reallocation of lipid metabolism from defense to growth. In the glutathione metabolism, four glutathione S-transferase genes (*GSTF14/16*, *GSTU16*, *RPP31*) were strongly upregulated (5–14 fold), whereas 15 other *GSTs* were downregulated by 55%–97% (Fig. S3D). This suggests that extract treatment may induce a certain degree of oxidative stress in rice. This selective regulation may reflect a targeted antioxidant response to mild oxidative stress induced by AQE, with upregulation of specific *GSTs* to scavenge oxidative damage and maintain glutathione homeostasis, while downregulation of non-essential *GSTs* conserves resources.

A total of 300 differentially expressed transcription factors (TFs) were identified, with 204 downregulated and 96 upregulated (Fig. S4). In the *WRKY* family, 42 TFs were downregulated and only 3 upregulated. Since *WRKY* TFs regulate disease resistance and abiotic stress responses, their downregulation suggests that AQE reduces defense-related transcriptional programs, redirecting resources toward growth. In the *bHLH* family, 15 TFs were upregulated and 18 downregulated; *bHLH* TFs are involved in abiotic stress signaling, development, and secondary metabolism. In the *GRF* family, five TFs were upregulated, consistent with enhanced cell division and leaf development, implying that AQE may promote tillering or vegetative growth. In the *ARF* family, only *OsARF13* was upregulated, possibly enhancing root development or cell elongation through auxin signaling and thereby improving nutrient uptake. *YABBY* genes *YAB3* and *YAB5* were upregulated, which are associated with plant height regulation. In the *C2H2* family, 8 TFs were upregulated and 11 downregulated; these TFs regulate auxin transport, architecture development, and disease resistance, and their differential expression may help balance resource allocation between growth and defense. Collectively, these results indicate that *Nostoc* sp. SCAU-13 extract reshapes the TF network by suppressing defense-related TFs and activating development-related TFs, thereby promoting growth and stress tolerance.

**Fig. 5:**
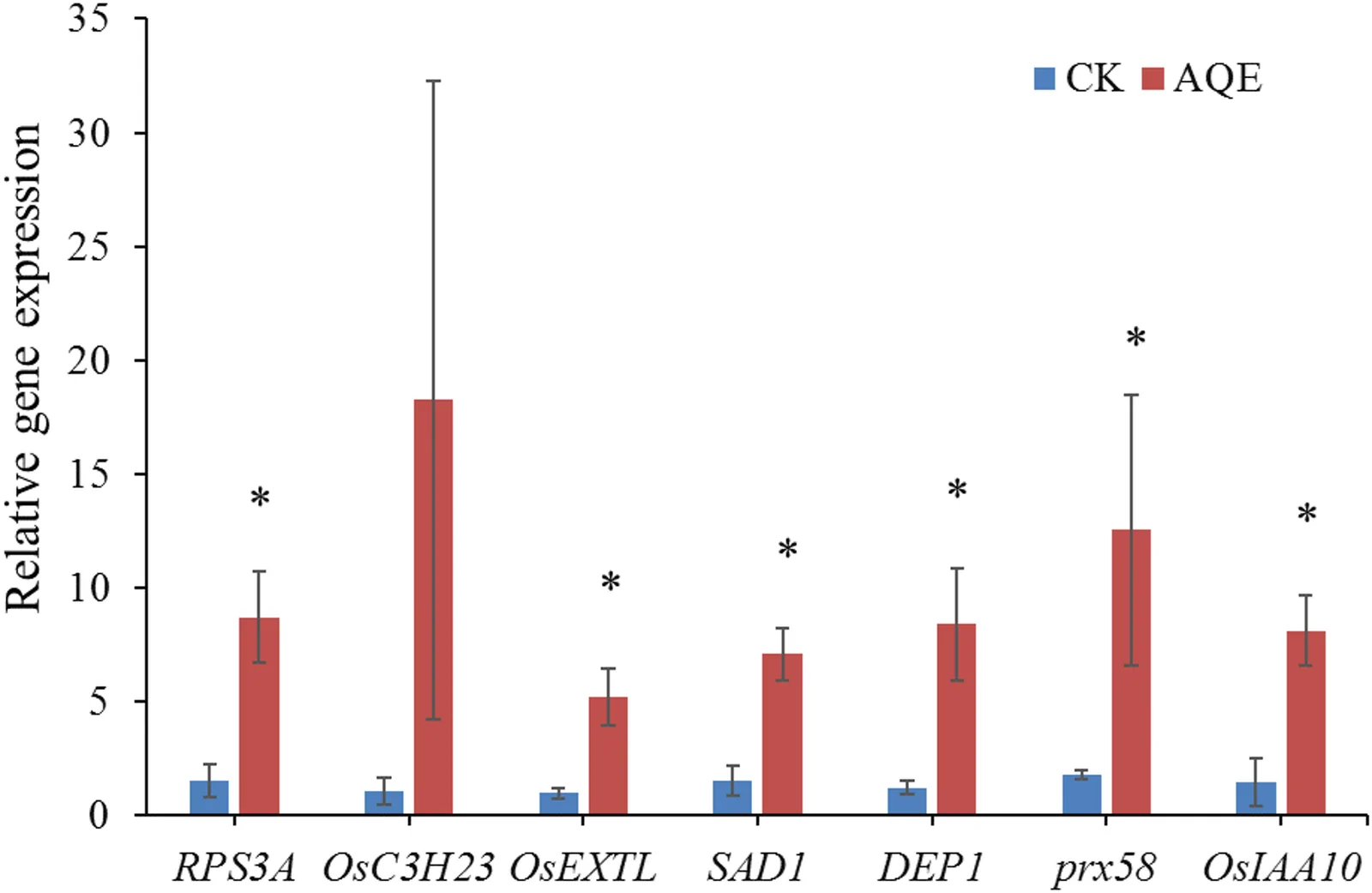
qRT-PCR validation of DEGs

The qRT-PCR validation of key genes (Fig. 5) confirmed their expression patterns revealed by RNA-seq.

### Rice field application experiment

#### Effects of cyanobacterial extract on rice stem morphology in the field

**Fig. 6:**
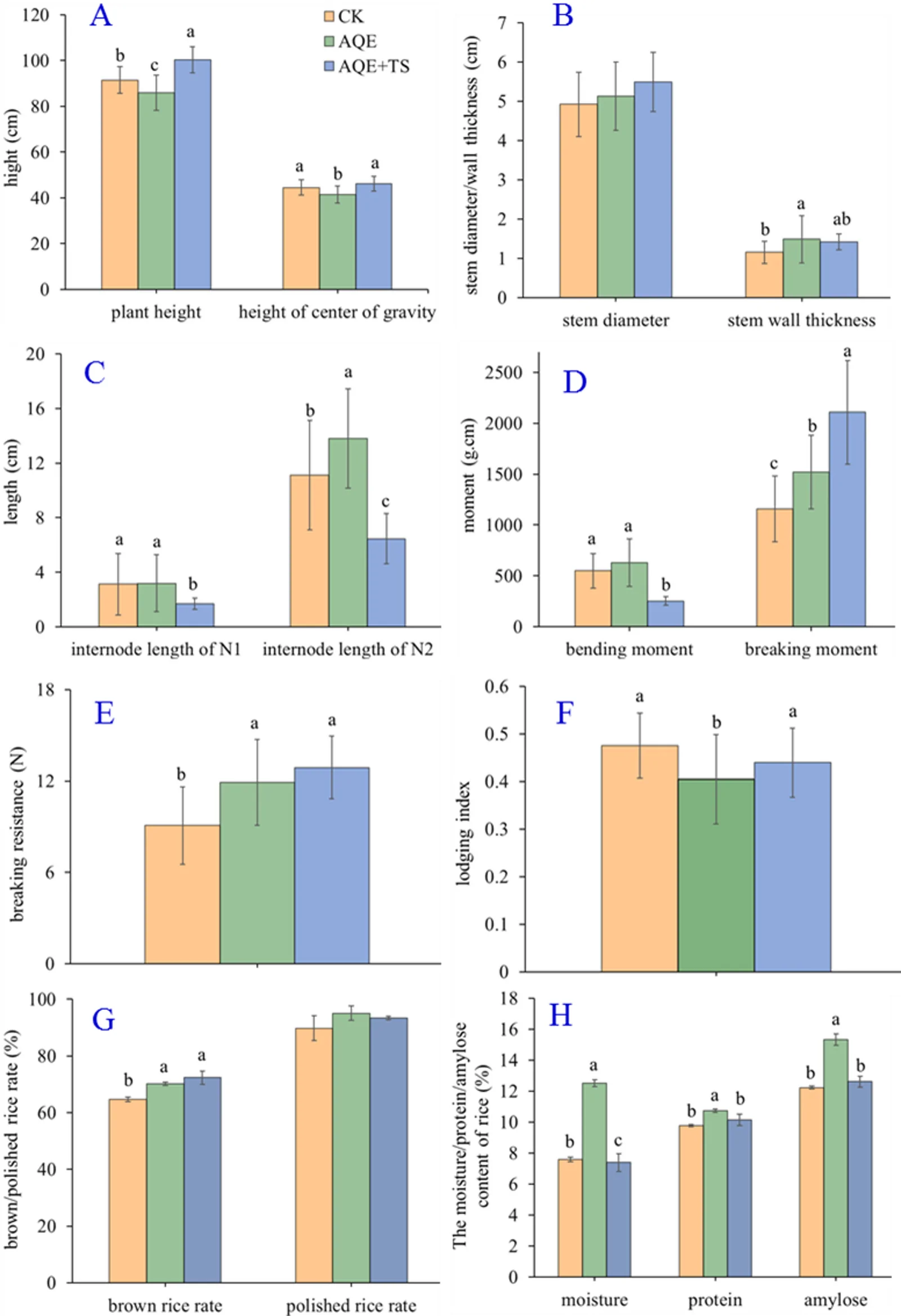
Field experiment results. **A** Plant height and height of center of gravity; (**B**) Stem diameter and wall thickness; (**C**) Internode length; (**D**) Bending moment and breaking moment; (**E**) Breaking resistance; (**F**) Lodging index; (**G**) Brown rice and milled rice rates; (**H**) Rice quality traits

Effects of AQE and AQE + TS on rice stem morphological traits in the field are shown in Fig. 6A and F. Plant height under AQE + TS was significantly greater than CK, and CK was significantly (*p* < 0.05) greater than AQE (Fig. 6A). Center of gravity height did not differ between AQE + TS and CK, but both were significantly higher than AQE. Stem diameter did not differ among treatments (Fig. 6B). Stem wall thickness under AQE was significantly greater than CK, and not significantly different from AQE + TS. The first internode (N1) length under AQE and CK was similar, and both were significantly longer than AQE + TS (Fig. 6C). The second internode (N2) length under AQE was significantly greater than CK, which was in turn significantly greater than AQE + TS. Bending moment did not differ between AQE and CK, but both were significantly higher than AQE + TS (Fig. 6D). The breaking moment under AQE + TS was significantly higher than AQE, which was significantly higher than CK. Breaking resistance under AQE + TS and AQE did not differ significantly, but both were significantly higher than CK (by 42% and 31%, respectively; Fig. 6E). However, the lodging index under AQE was significantly lower than both CK and AQE + TS (reduced by 15% relative to CK), with no significant difference between the latter two (Fig. 6F).

#### Effects of cyanobacterial extract on rice yield and quality in the field

As shown in Table 3, the effective panicle number was highest under AQE + TS, but differences were not significant. Grain number per panicle under AQE and AQE + TS significantly increased by 40% and 24.6% over CK, respectively. Filled grain number under AQE and AQE + TS was significantly higher than CK (by 27% and 35%, respectively). Thousand-grain weight and filled grain yield under AQE + TS were significantly greater than both CK and AQE, which did not differ from each other. Milling quality is shown in Fig. 6G: the brown rice rate under AQE + TS (72%) was significantly higher than CK (64%), whereas milled rice rate did not differ among treatments. Grain quality results are shown in Fig. 6H: moisture content under AQE was significantly higher than CK, and CK was significantly higher than AQE + TS. Both protein content and amylose content under AQE were significantly higher than CK and AQE + TS.

**Table 3:** Effects of adding extract and active cyanobacterium TS on rice yield in field experiment

Treatment	Effective number of panicles	Number of grains per panicle	Number of filled grains	Thousand grain weight (g)	Total yield (t/ha)
CK	11 ± 2	126 ± 38b	111 ± 35b	17.5 ± 0.4b	6.51 ± 1.25b
AQE	10 ± 1	177 ± 61a	141 ± 43a	15.2 ± 4.6b	6.28 ± 0.19b
AQE + TS	12 ± 2	157 ± 59a	150 ± 59a	19.8 ± 0.8a	9.36 ± 0.93a

different letters in the same column indicate significant differences (*p* < 0.05)

## Discussion

### Agricultural application of nitrogen-fixing cyanobacteria

The agricultural use of nitrogen-fixing cyanobacteria began in 1939, when they were first reported as a biofertilizer in India. Since then, this practice has gained international attention. Numerous studies have demonstrated that inoculating paddy fields with nitrogen-fixing cyanobacteria increases soil nitrogen content, leading to significant and stable yield gains while reducing chemical nitrogen fertilizer inputs [1, 23]. In China, research flourished in the 1950–1960 s and confirmed their yield-promoting effects in rice [16], but large-scale adoption did not occur, likely due to the availability of inexpensive synthetic nitrogen fertilizers at that time. Nitrogen-fixing cyanobacteria have multiple agronomic functions: in addition to nitrogen fixation, they can increase soil organic matter, solubilize phosphorus and potassium, oxygenate the root zone, and secrete growth-promoting substances [26, 40]. Recent studies show that substituting nitrogen fertilizer with nitrogen-fixing cyanobacteria can reduce nitrogen leaching in red paddy soils, and combined application with chemical fertilizers may improve nitrogen management [31, 32]. Our previous work confirmed that indigenous nitrogen-fixing cyanobacteria can increase rice yield while reducing nitrogen input [4]. Their role in inducing disease resistance has also been reported [25]; for example, certain cyanobacterial strains can suppress rice sheath blight [34]. *Anabaena* treatment stimulates tomato growth and enhances resistance to *Fusarium wilt*, accompanied by increased activity of defense-related enzymes [26]; *Calothrix* sp. and *A. laxa* can induce antibacterial capacity in seedlings [15]; and aqueous extracts of *Anabaena* elicit systemic resistance against powdery mildew in zucchini [27]. The present study further reveals that extracts from nitrogen-fixing cyanobacteria can enhance lodging resistance in rice. Collectively, these findings highlight the multiple benefits, economic and environmental advantages, and promising prospects of nitrogen-fixing cyanobacteria in agriculture.

### Mechanism of lodging resistance in rice induced by nitrogen-fixing cyanobacterial extracts

Lodging resistance in rice largely depends on the physical properties and chemical composition of the culm. Physically, the length of basal internodes, stem diameter, wall thickness, and degree of filling are closely associated with culm mechanical strength and are key determinants of lodging [21, 42]. However, improving lodging resistance must consider biomass partitioning. Simply increasing stem diameter and wall thickness can enhance strength, but excessive structural reinforcement may divert photoassimilates to vegetative organs at the expense of grain yield, creating a trade-off between lodging resistance and yield [24]. The content of non-structural carbohydrates (NSC) in basal internodes, together with the major cell wall components—lignin, cellulose, hemicellulose, and silica—collectively determine culm mechanical strength and rigidity [20]. The cell wall is composed of primary and secondary walls [41]. The degree of lignification and cellulose deposition density in sclerenchyma cells is directly linked to their mechanical support function [29]. Culm compressive strength correlates positively with lignin content, and mechanical support correlates with cellulose content and density [17, 38]. Thus, modulating biochemical traits such as lignin and cellulose content is a key strategy to improve culm strength and lodging resistance.

This study demonstrated that AQE from *Nostoc* sp. SCAU-13 significantly enhanced lodging resistance in rice. Integrating extract composition, biochemical, and omics data, the underlying mechanism may involve: (1) Optimization of culm morphology and physical structure. AQE significantly increased stem diameter, wall thickness, and breaking resistance, while reducing N2 internode length. These morphological changes may be attributed to plant growth regulators in AQE (e.g., IAA, abscisic acid, oleamide), which could inhibit excessive internode elongation by antagonizing gibberellin signaling (consistent with upregulation of *YABBY* and *GRF* genes in the transcriptome) and promote stem thickening. (2) Modification of culm chemical composition and enhancement of cell wall strength. AQE significantly increased lignin content (14.9% over CK) and upregulated key genes involved in stem development and secondary cell wall formation, such as *OsNAC5*,* OsIDD4*, and *GRF1*. *OsNAC5* has been reported to activate CCR10 and promote lignin synthesis [3], while *OsIDD4* and *GRF1* are involved in stem growth regulation [14]. The upregulation of these genes collectively explains the increased stem diameter and lignin deposition. Moreover, *OsTB1* can directly regulate lignin biosynthesis genes, enhancing culm strength and lodging resistance [33]. Increased NSC content may also synergistically improve rigidity by contributing to cell wall filling and maintaining turgor pressure [2]. Phytohormones in AQE may play important roles in regulating lignin synthesis [19], and AQE treatment may achieve a favorable balance between structural and non-structural carbohydrates [5]. (3) Supply of bioactive components that modulate metabolic and stress pathways. AQE is rich in bioactive substances, including antioxidants such as glutathione metabolites, polyphenols, and lipoic acid, which may scavenge reactive oxygen species and maintain cellular homeostasis. Interestingly, unlike the typical induction of antioxidant enzyme genes by microbial elicitors, GO analysis revealed downregulation of defense response genes, suggesting that rice did not perceive AQE as a stress factor. Some metabolites may directly promote lignin deposition through the phenylpropanoid pathway. Terpenoids may influence stem development by modulating gibberellin signaling (e.g., *OsGA20ox*). Abscisic acid and related compounds directly participate in stress responses [39]. AQE also induced differential expression of TFs such as *WRKY* and *bZIP* (e.g., downregulation of *OsWRKY30*), which may indirectly enhance culm toughness. We hypothesize that upregulation of *OsNAC5* serves as a key molecular node linking cyanobacterial extract components, lignin synthesis, and the lodging resistance phenotype [3]. Furthermore, among the genes validated by qRT-PCR, *OsEXTL* has been shown to increase cellulose content, stalk strength, and lodging resistance by modulating cell elongation and cell wall thickening without compromising grain yield or biomass [9].

Overall, these results indicate that bioactive extracts from nitrogen-fixing cyanobacteria enhance rice lodging resistance through multidimensional and multitarget effects: at the physical level, by optimizing culm morphology and improving mechanical strength; at the chemical level, by increasing structural compounds and NSC reserves to reinforce cell walls; and at the molecular level, by supplying diverse bioactive substances that regulate key gene expression, activate phenylpropanoid metabolism, and promote secondary cell wall formation and antioxidant defense.

Notably, in the field trial, AQE significantly increased grains per panicle and filled grain number, but total yield did not differ significantly from CK—in contrast to the pot experiment where AQE significantly increased yield. This discrepancy may stem from more complex and variable environmental conditions in the field, and possibly from a biological trade-off involving the transcriptional repression of jasmonic acid and defense-related pathways under natural pathogen pressure. Nevertheless, the positive effect of AQE on lodging resistance was clearly validated in the field.

Moreover, the combined application of AQE and live TS—a strain that strongly promotes yield but increases lodging risk—significantly improved yield and thousand-grain weight while simultaneously enhancing culm strength, thereby offsetting the lodging tendency. This biostimulant-based strategy (using nitrogen-fixing cyanobacterial extracts) offers distinct advantages over traditional resistance breeding or chemical regulation: it is environmentally friendly, sustainable, free of genetic modification concerns, and can synergistically improve yield, highlighting its great agricultural potential. Nevertheless, this study has limitations. The relatively small field trial area and limited harvest sampling may amplify microenvironmental heterogeneity and affect the representativeness of yield and related data. Future studies will expand the experimental and sampling areas to further validate the efficacy of this technology.

## Conclusion

This study identified the extract of *Nostoc* sp. SCAU-13 as the most effective for inducing lodging resistance in rice and optimized the extraction process. Field experiments demonstrated that the combined application of the cyanobacterial extract (AQE) and the high-yield-promoting live cyanobacterium *Constrictifilum* sp. TS increased rice yield while maintaining lodging resistance, providing a green strategy to mitigate the lodging risk associated with large-scale use of TS. Analysis of the extract composition, together with biochemical and transcriptomic data, suggests that the active compounds in the extract induce lodging resistance through multidimensional and multitarget effects. This is the first study to employ multi-omics approaches to elucidate the mechanism by which nitrogen-fixing *Nostoc* sp. extracts enhance lodging resistance in rice. Bioactive substances derived from nitrogen-fixing cyanobacteria are renewable and therefore offer considerable environmental benefits and sustainable development potential.

## Supplementary Information

Supplementary Material 1.

Supplementary Material 2.

## Data Availability

The raw transcriptome data of of rice has been saved in the National Bioinformatics Center of China under BioProject accession number PRJCA055729 (GSA: CRA037049) and can be accessed through https://ngdc.cncb.ac.cn/gsa/browse/CRA037049.
