# Supplementary material for: Nostoc sp. extract enhances rice lodging resistance by remodeling the gene expression network: mechanism and field application
Source: BMC Plant Biol. 2026 Jul 15;26:1734. doi: 10.1186/s12870-026-09546-y (PMC13651268; doi:10.1186/s12870-026-09546-y)
Supplement: Supplementary file 1 [file 12870_2026_9546_MOESM1_ESM.docx]

Fig. S1 The impact of algal extracts obtained by different extraction methods on rice.

**A**

**B**

Fig. S2 Pie chart of primary classification of metabolites in positive (A) and negative (B) ion modes

**A**

**B**

**C**

**D**

Fig. S3 KEGG enrichment pathway (A: ribosome, B: phenylpropanoid biosynthesis, C: linoleic acid metabolism, D: glutathione metabolism)

Fig. S4 Differentially expressed transcription factor family (red bars indicate upregulation, blue bars indicate downregulation)
